# Serum Cytokeratin 18 M30 Levels in Chronic Hepatitis B Reflect Both Phase and Histological Activities of Disease

**DOI:** 10.1155/2017/3480234

**Published:** 2017-07-30

**Authors:** Magdalena Świderska, Jerzy Jaroszewicz, Anna Parfieniuk-Kowerda, Magdalena Rogalska-Płońska, Agnieszka Stawicka, Anatol Panasiuk, Robert Flisiak

**Affiliations:** ^1^Department of Infectious Diseases and Hepatology, Medical University of Bialystok, Bialystok, Poland; ^2^Department of Physiology, Medical University of Bialystok, Bialystok, Poland; ^3^Department of Infectious Diseases and Hepatology in Bytom, Medical University of Silesia, Katowice, Poland

## Abstract

Chronic hepatitis B has highly a dynamic course with significant fluctuations of HBV-DNA and ALT impeding assessment of disease activity. New biomarkers of inflammatory versus noninflammatory stages of HBV infection are urgently needed. Cytokeratin 18 epitope M30 (M30 CK-18) is a sensitive marker of cell death. We aimed to investigate an association between serum M30 CK-18 and histological activity and phase of HBV infection. 150 Caucasian patients with HBV-infection were included in the study. Serum M30 CK-18 levels reflected phase of disease, being significantly higher in both HBeAg(+) and HBeAg(−) hepatitis B in comparison to HBsAg(+) carrier groups. The highest serum M30 CK-18 levels were observed in subjects with the most advanced stages of HBV. Moreover, its serum concentrations correlated with both inflammatory activity and fibrosis advancement (ANOVA *P* < 0.001). Importantly, serum M30 CK-18 levels were able to discriminate patients with mild versus moderate-advanced fibrosis (AUC: 0.86) and mild versus active liver inflammation (AUC: 0.79). M30 CK-18 serum concentration has good sensitivity and specificity in discriminating mild versus moderate/severe fibrosis and inflammation even in patients with normal ALT activity. This study suggests M30 CK-18 as a potential noninvasive marker of disease activity and also a marker of phase of persistent HBV infection.

## 1. Introduction

Persistent HBV infection, especially following HBeAg seroconversion, is a highly dynamic disease with significant fluctuations of HBV-DNA which may be observed in every phase of disease [1]. This variability is a result of a complex interplay between viral factors, mainly quantity and transcriptional activity of cccDNA, and strength of HBV-specific immune responses. It is important to note that frequent exacerbations of chronic hepatitis B (CHB) results in the progression of liver fibrosis which eventually leads to liver cirrhosis [2]. The commonly used marker of inflammatory activity, ALT, has significant limitations in HBeAg(−) hepatitis. It has been shown that as many as 44% of HBeAg(−) hepatitis ALT remain within normal range for the majority of time despite of disease progression. Importantly, its increase could only be observed once a year in almost 60% of those patients [3]. Moreover, ALT activity does not reflect inflammatory activity in the liver nor extent of fibrosis in CHB. The presence of biopsy-proven histologic damage (necroinflammation and fibrosis) is common even when ALT is less than 2XULN in patients with chronic hepatitis B (CHB) [4]. Liver biopsy has been a gold standard of viral hepatitis activity; however, this invasive procedure is associated with risk of complications and yields variability due to the uneven distribution of lesions [5]. Liver biopsy examination shows interpathologist variations ranging from 10 to 20% [6]. Finally, novel noninvasive modalities including elastography show limited capability of differentiating between mild and moderate fibrosis, which is of importance for the selection of anti-HBV therapy candidates [7]. A potential advantage of serum noninvasive markers of liver fibrosis is that total liver fibrosis is reflected consequently reducing the risk of intra- and interassay variability which is known for liver biopsy and liver elastography. Therefore, there is a strong need for new noninvasive markers of liver injury, especially detecting inflammatory activity not only fibrosis, particularly at less advanced stages. Knowing that cell death by apoptosis is an important step for the development of CHB, the integration of markers of cell death appears to be justified [8, 9].

Apoptosis is a major cause of hepatocytes' death in chronic viral hepatitis. Apoptosis leads to the activation of several cysteine-aspartate proteases, called caspases. Caspases cleave cellular proteins, including cytokeratin-18 (CK-18). CK-18 is an intermediate filament protein of the cytoskeleton which may be found in epithelial cells, especially of the digestive tract. Along with cytokeratin 8, CK-18 is the only cytokeratin found in the hepatocytes [10]. Recently, the evaluation of CK 18 has been validated as a marker of inflammation activity and fibrosis in chronic hepatitis C (CHC) and nonalcoholic fatty liver disease (NAFLD) [11–14]. During apoptosis, activated caspases 3, 6, 7, and 9 are able to cleave cytokeratin-18 at specific peptide recognition sites [12, 15]. As a result of caspase cleavage, cytokeratin-18 is cut at position 387 to 396 and releases its fragment M30. Epitope M30 is released into the bloodstream as a result of cell death; therefore, it is possible to use M30 CK-18 as a circulating biomarker of epithelium apoptosis [14, 16]. Previously, Papatheodoridis et al. [17] demonstrated significantly higher concentrations of M30 CK-18 in patients with active CHC-B compared to nonactive carriers with low HBV replication. Of importance, serum concentration of CK-18 correlated with HBV replication in contrast to chronic hepatitis C. There was also a significantly higher concentration of M30 CK-18 in patients with CHC-B and normal ALT activity compared with HBsAg carriers [17, 18].

Another recent marker in CHB is serum HBsAg quantification which reflects the phase of HBV infection [19]. In HBV-genotype D, the combined single-time measurement of HBV-DNA < 2000 IU/mL and HBsAg < 1000 IU/mL had excellent diagnostic accuracy in discriminating active and inactive HBV persistent infection in the long-term follow-up [20]. More recent data suggested that HBV genotype should be included as another factor influencing HBsAg levels in the natural course of CHB and the correlation between phase of disease and HBsAg levels might be weak/not present in HBV-A. Importantly, an association between HBsAg and liver fibrosis stage has been mainly shown in patients with HBeAg(+) disease and its significance decreases in liver cirrhosis, especially in HBeAg(−) [21].

The aim of study was to evaluate the clinical usefulness of serum M30 CK-18 in the noninvasive assessment of phase of disease, inflammatory activity, and liver fibrosis in the natural course of chronic hepatitis B in predominantly HBV genotype A infected population.

## 2. Patients and Methods

150 Caucasian patients with persistent HBV infection (median age 33, 86 males) were included in this single-center, cross-sectional study. All patients were adults and had HBsAg(+) for at least 6 months. The clinical characteristics of the studied population is presented in Table 1. HBeAg-negative subjects were further stratified into low replicative carriers (LRC: normal ALT-activity and HBV-DNA < 2000 IU/mL), high replicative carriers (HRC: HBV-DNA between 2000 and 20,000 IU/mL, normal ALT (N ALT) and/or no inflammatory lesions in liver biopsy), and HBeAg-negative hepatitis (ENH: HBV-DNA > 20,000 IU/mL and increased ALT and/or inflammatory lesions in liver biopsy). Liver biopsies were performed only in patients with HBV-DNA ≥ 2000 IU/mL and clinical suspicion of active hepatitis B, as a part of the qualification for the anti-HBV therapy. The results of liver biopsy were available in 66 (44%) patients. Exclusion criteria included coinfection with HCV, HIV, liver steatosis, autoimmune disorders, malignancies, and current alcohol abuse. The study protocol was approved by the Bioethics Committee of Medical University in Bialystok, and informed consent was obtained from each participant.

### 2.1. Liver Morphology

Liver tissue was collected from the right liver lobe, using the Menghini method with a disposable set of Hepafix Luer Lock (Braun) 16G needle. Tissue was fixed with 4% formalin and then paraffin embedded. Biopsies' length was in the range of 10–30 mm. Samples sections were stained with: hematoxylin-eosin to identify liver inflammation and Sirius Red for fibrosis assessment. Liver biopsies from CHB patients were scored by a blinded pathologist using 5-category (grading of 0–4 and staging of 0–4) Scheuer classification.

### 2.2. Serum M30 CK-18 Measurement

A total volume of 10 mL peripheral venous blood was taken at the time of liver biopsy and/or liver function test assessment and stored at −80°C until further processing. Serum M30 CK-18 was measured by M30-Apoptosense ELISA assay (PEVIVA AB, Bromma, Sweden) according to the manufacturer's recommendations. Lower limit of detection and intra- and interassay variability were (25 U/L (8.44 pg/mL), <10%, resp.).

### 2.3. Serum HBsAg and HBV-DNA Quantification

Serum HBsAg levels were measured using the Abbott ARCHITECT® assay (Abbott Diagnostics, Abbott Park, IL). Dynamic range of this test was 0.05–250 IU/mL. Dilution of samples was 1 : 20, 1 : 100, and 1 : 500, respectively. HBsAg result less than 0.05 IU/mL was measured without dilution. HBsAg quantification is expressed in IU/mL. Serum HBV-DNA was measured using COBAS AmpliPrep/COBAS TaqMan (Roche Diagnostics, Mannheim, Germany) and TaqMan Universal Master Mix (Applied Biosystems, Foster City, CA) with detection limits of 12 IU/mL and 50 IU/mL, respectively. Results are given in IU/mL. HBsAg and HBV-DNA values were log-transformed.

### 2.4. Statistical Analyses

Data is presented as median (IQR) and percentage when appropriate. Nonparametric, distribution-free tests were applied. Differences between groups were analyzed by Mann–Whitney *U* test and the Kruskall-Wallis ANOVA test. Correlation analyses were performed by Spearman's test. In order to determine the diagnostic accuracy (sensitivity and specificity) of M30 CK-18, receiver operating characteristic (ROC) curves were drawn and the area under the curve (AUC) was calculated. A *P* < 0.05 was considered statistically significant. Statistical analyses were performed by the GraphPad Prism5 (La Jolla, CA) and Statistica 10.0 (Statsoft, Tulsa, OK).

## 3. Results

In this Caucasian treatment-naïve cohort, among the 150 subjects included, 93% were HBeAg-negative and 69% had normal ALT activity. Among the HBeAg(−) patients, 47 were low replicative carriers (LRC), 28 high replicative carriers (HRC), and 65 HBeAg(−) hepatitis subjects (ENH). Importantly, among the ENH patients with available liver biopsies in 11 (28%), ALT activity was within normal range although active inflammatory lesions (G2 or more) were shown in the liver biopsy (Table 1).

### 3.1. M30 CK-18 and Biochemical Activity and Phase of Persistent HBV Infection

**Figure 1:**
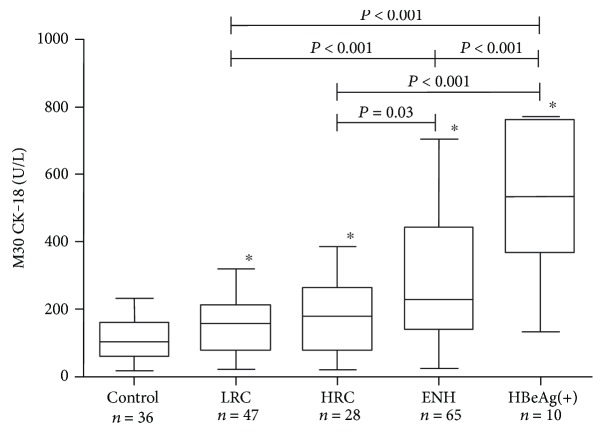
Median (IQR) serum M30 CK-18 levels in different phases of persistent HBV infection (LRC: low replicative carriers; HRC: high replicative carriers; ENH: HbeAg(−) hepatitis B; HBeAg(+): HBeAg(+) immune clearance phase). ^∗^*P* < 0.05 in comparison with the control group. All comparisons by use of Mann–Whitney *U* test.

Serum M30 CK-18 correlated with ALT (*R* = 0.28, *P* = 0.004), GGT activity (*R* = 0.24, *P* = 0.005), and platelet count (*R* = −0.18, *P* = 0.05) (Table 2). Subjects with increased ALT had significantly higher serum M30 CK-18 compared to the group with normal ALT (267 (129–567) versus 163 (109–244) U/L, *P* = 0.001). Moreover, M30 CK-18 showed a significant association with HBV-DNA (*R* = 0.26, *P* = 0.02), which was further proved in multivariate regression (Table 2). Serum M30 CK-18 levels showed an association with phase of disease. The lowest values were observed in inactive CHB (LRC: 156 (81–208) IU/mL; HRC: 177 (78–261) IU/mL), while M30 CK-18 was significantly higher in both active hepatitis B groups (ENH: 225 (141–438) IU/mL; HBeAg(+) hepatitis 532 (367–759) IU/mL), Figure 1().

### 3.2. M30 CK-18 and Histological Activity of Hepatitis

**Figure 2:**
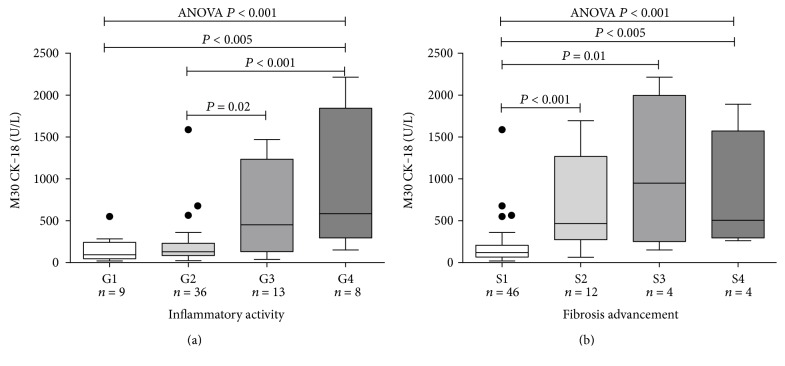
Median (25–75% CI) serum M30 CK-18 (U/L) concentration in HBV-infected patients with subsequent grades of inflammatory activity (a) and stages of liver fibrosis (b) in the liver histology. Comparisons by use of Mann–Whitney *U* test and Kruskal-Wallis ANOVA.

**Figure 3:**
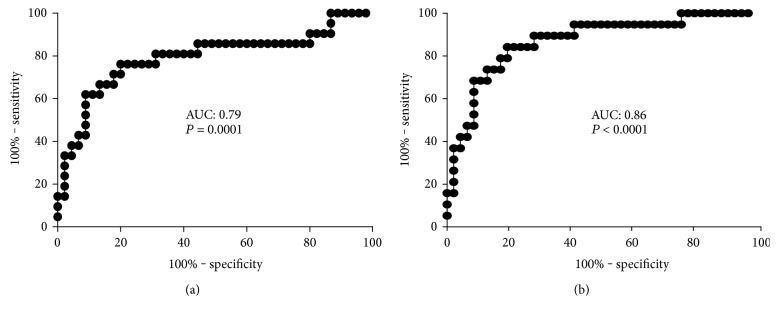
ROC curves for serum M30 CK-18 concentrations as a marker of (a) active liver inflammation (G1-G2 versus G3-G4) and (b) advanced fibrosis (S0-S1 versus S2–S4). AUC: area under curve.

Serum M30 CK-18 levels were highly associated with the histological advancement of liver fibrosis (ANOVA, *P* < 0.0001) and the degree of inflammation (ANOVA, *P* = 0.0009) (Figure 2(), Table 3). Serum M30 CK-18 was more than trifold higher in patients with moderate/severe (S2–S4) versus mild (S1) fibrosis (534 (272–1345) versus 119 (67–207) IU/mL, *P* < 0.0001) (Table 3). To determine the diagnostic potential of the M30 CK-18, ROC analysis was performed. ROC showed good a discriminatory ratio for patients with moderate/severe versus mild fibrosis (AUC: 0.86, *P* < 0.0001), with 84% sensitivity and 80% specificity for M30 CK-18 value of 253 IU/mL (Figure 3()). Similarly, M30 CK-18 was significantly higher in subjects with active inflammation (G1-G2: 124 (74–229) versus G3-G4: 466 (229–1307) IU/mL, *P* < 0.001) with AUC of 0.79 (Figure 2()).

### 3.3. HBsAg Levels and the Phase of Persistent HBV Infection

As reported previously, HBsAg serum levels showed only moderate correlation with HBV-DNA (*R* = 0.17, *P* = 0.03) and were not associated with ALT or fibrosis stage in the natural history of HBeAg(−) persistent HBV infection [19]. The highest concentrations of serum HBsAg were observed in both phases of active hepatitis with positive HBeAg (4259 (3898–4960) log10 IU/mL) and negative HBeAg (4252 (4077–4618) log10 IU/mL). Patients with low disease activity (HBsAg carriers) had lower HBsAg concentrations (HRC: 4093 (3623–4631) log10 IU/mL; LRC: 4085 (3277–4598) log10 IU/mL), with statistically significant difference between ENH and low replicative HBsAg carriers (*P* = 0.03). Serum CK-18M30 did not show an association with HBsAg concentration (*R* = 0.04, *P* = 0.61), which suggests an additional advantage of M30 CK-18 measurement in addition to HBsAg quantification.

## 4. Discussion

In this study, we evaluated an association between serum M30 CK-18 and biochemical and histological activities of chronic hepatitis B. Establishing a more precise marker of both disease activity and fibrosis in CHB currently unmet the medical needs, especially in HBeAg(−) hepatitis which is characterized by significant variations of ALT and HBV-DNA. Such noninvasive biomarker would allow precise dissection between inflammatory and noninflammatory stages of CHB but also selection of best candidates for PEG-IFN and novel immunomodulatory therapies [22].

Previous studies on M30 CK-18 have generally focused on CHC and NAFLD [14]. Increase in M30 activity has been shown in patients with CHC [13, 17, 23]. Bantel et al. [13] proved that 27% of patients with CHC normal level of aminotransferases occurred despite developing liver damage. Moreover, the authors found higher concentrations of M30 CK-18 in patients with advanced fibrosis and suggested that M30 might be more sensitive than aminotransferase for identifying liver injury in CHC [13]. In another study by Papatheodoridis, a positive correlation between serum CK-18 and severity of inflammatory activity and fibrosis was found in chronic HCV infection. In this study, the concentration of CK-18 exceeding 225 U/L demonstrated a positive and negative predictive values for moderate–severe histologic lesions in the liver biopsy of 70% and 74% [18]. Similar findings by Parfieniuk-Kowerda et al. have been shown in our center [24]. The ROC analysis revealed that serum M30 CK-18 showed 75% sensitivity and 75% specificity in differentiating between mild, moderate, and severe inflammation at a concentration of >204 U/L, whereas higher concentration (>330 U/L) achieved 89% sensitivity and 78% specificity between mild and moderate to advanced liver fibrosis in chronic hepatitis C [24].

Likewise, in NAFLD, serum M30 CK-18 was shown to be elevated and strongly associated with ALT and AST activities. A negative correlation between the level of M30 and AST/ALT ratio was observed suggesting that apoptosis was more a dominant mechanism of cell death [25]. In another study, a novel algorithm for assessing fibrosis in NAFLD was suggested, including ALT, AST, M30, M60, and HA. Importantly, the authors suggested M30 and M65 as more important for the decision than the classic liver parameters [9]. In further study in NAFLD, apoptosis-specific M30 CK-18 correlated with reticuloendothelial system (RES) cell iron in the liver and nonalcoholic steatohepatitis [10]. In their recent study, In their recent study, Bantel et al. applied an improved ELISA for serum cytokeratin-18 fragment detection suggesting the use of the method to evaluate the early stages of development of NASH; it also enables distinguishing differences between patients with a minimal (≤10%) and advanced (>10%) hepatic steatosis [26].

There is limited information concerning M30 CK-18 levels in CHB patients. Compared with conventional indicators of the activity of liver disease, determination of caspase activity, expressed as the concentration of CK-18 in the serum, may be a more sensitive method for assessing the activity of persistent HBV infection. The results of our study show that there is a significant difference in the concentration of M30 CK-18 between consecutive phases of CHB including HBeAg (+), LRC, HRC, and ENH (ANOVA, *P* < 0.0001). Interestingly, this marker was able to differentiate patients with active HBeAg-negative hepatitis B and low replicative and high replicative carriers. It is most likely a consequence of association between M30 CK-18 and ALT activity but also with HBV-DNA found in our study. A positive correlation between serum M30 CK-18 and ALT-activity was found in all previous studies in CHB [14, 17, 27], while with HBV-DNA, only in some [17]. This might be the effect of study group composition where all phases of CHB should be present to allow appropriate comparisons. Importantly, also site-specific phosphorylation of K18 correlated with the elevation of both histological lesions and enzymatic activities of alanine aminotransferase in CHB which further supports biochemical data [28].

Definitely in the era of noninvasive methods of activity assessment, the most important is the association between serum M30 CK-18 and histologic advancement of lesions in the liver in CHB. We have demonstrated that serum concentrations of M30 CK-18 were associated with histological inflammatory activity (ANOVA, *P* = 0.0009) and advancement of liver fibrosis (ANOVA, *P* < 0.0001). It is important since majority of the previously available noninvasive tests, including transient elastography, are not able to differentiate between the degrees of inflammation in CHB, especially in the case of the less advanced liver damage. ROC curve analysis showed that the single measurement of the concentration of M30 CK-18 > 253 U/L differentiates patients with mild to moderate versus active hepatitis with 80% specificity and 76% sensitivity. Furthermore, M30 CK-18 with the same threshold exhibited 84% sensitivity and 80% specificity in differentiating patients with mild versus advanced fibrosis (S1 versus S2–S4). Naturally, restricted number of patients with histological evaluation of liver inflammation and fibrosis is a limitation of the study. Liver biopsies, as an invasive procedure, were only performed in subjects with significant HBV viral load. On the other hand, even in this limited sample study, statistical results suggest for a highly significant association between serum M30 CK-18 and inflammatory activity as well fibrosis stage. In the previous study, Sumer et al. reported [14] that the M30 CK-18 levels are the highest in patients with liver cirrhosis. Also, Bae et al. [27] found that serum M30 levels are associated with the presence of significant inflammation. In our study, by means of serum M30 CK-18, the differentiation even between mild (S1) and moderate–advanced (S2–S4) fibrosis was possible. This marker could be especially useful in subjects with normal ALT and slightly elevated HBV-DNA as well as in HBeAg(+) highly vireamic patients with normal ALT in which otherwise long-term follow-up or liver biopsy would be necessary in order to assess the activity of the disease.

Clinical usefulness of serum M30 CK-18 could potentially be further increased by combining it with already established serological markers of hepatitis B, including HBsAg quantification. The concentration of HBsAg in serum reflects the transcriptional activity of the cccDNA and the degree of integration of HBV into the host genome [29]. In our previous study including 226 Caucasian patients with chronic hepatitis B, HBsAg levels showed significant differences during the natural course of HBV infection. Low HBsAg levels were characteristics of inactive carriers and differentiated this group from HBeAg(−) hepatitis B patients with normal ALT activity and fluctuating HBV-DNA [19]. This finding was further confirmed by Brunetto et al. who showed that single time-point, combined measurement of HBsAg < 1000 IU/mL and HBV-DNA < 2000 IU/mL identified inactive HBsAg carriers with 94.3% diagnostic accuracy in HBV genotype D infection [20]. Importantly, HBsAg levels and on-therapy dynamics of decline depends on HBV genotype which has to be taken into account. In the current study, again, we showed higher HBsAg concentrations in patients with HBeAg-negative hepatitis compared to patients with low replication (4.252 versus 4.085 log10 IU/mL, *P* = 0.04). The difference was less pronounced than in the previous study [19] which most likely results from high concentrations of HBsAg observed in the serum of patients infected with genotype A, which dominates the population of Poland (>70%) [30]. There was no correlation between serum HBsAg and M30 CK-18 levels, which was expected since HBsAg levels do not correlate with inflammatory activity in CHB. On the other hand, the combined measurement of those two markers, M30 CK-18 (inflammatory activity and fibrosis) and HBsAg-levels (cccDNA activity and phase of disease), could deliver precise characteristics of CHB activity.

Majority of HCC cases are related to chronically infected HCV (75–80%) and HBV (10–15%). An important risk factor for HCC is cirrhosis of the liver (80–90% of HCC cases) [31]. Recent studies have shown that elevated M30 CK-18 concentrations may be a useful marker for early stages HCC. Elalfy et al. calculated a ROC curve for the M30 CK-18 to discriminate between macrovascular invasions of HCC, which has shown 100% sensitivity and 98% specificity for a cutoff of 304.5 ng/mL (AUC: 0.997, *P* < 0.001) [32].

For noninvasive evaluation of fibrosis and necroinflammatory activity, several serum biomarkers can be applied, including Fibrotest and Actitest, Hepascore MPP. Ngo et al. [33] in a large group of 1300 patients with CHB showed higher Fibrotest prognostic value for ALT in predicting disease progression [33]. Fibrotest and Actitest and HBV-DNA combinations have been shown to better separation patients with low HBV replication as compared to the measurement ALT or HBV-DNA alone [33]. Despite the large study group (1300 patients), comparing Fibrotest and Actitest of a liver biopsy was performed only in the group of 97 patients. Hepascore is another test evaluation for histological changes in the liver. The comparison of Hepascore to Fibrotest shows a similar diagnostic value with high AUC (area under the curve) values for significant fibrosis, advanced fibrosis, and cirrhosis [34]. The abovementioned studies were designed primarily to assess the degree of fibrosis in hepatitis C, and their utility in evaluating fibrosis in CHB was often not validated. Another study which noninvasively assessed fibrosis shows that the overall rate was significantly lower in patients with CHB compared to patients with CHC in the early stages of fibrosis (*F* ≤ 2) [8]. It is also important to underline that panels of biomarkers like Fibrotest and Actitest which are composed of several complex measures (e.g., alpha-2 macroglobulin, haptoglobin, apolipoprotein A1, total bilirubin, GGT, ALT, and age) and are also patented which increases their costs in routine clinical practices.

Another noninvasive marker of liver fibrosis may be MMPs which are the main enzyme-degrading proteins of extracellular matrix and play an important role in tissue reconstruction and repair in physiological and pathological conditions. Previous studies have shown that serum MMP-2 levels were statistically higher in CHB patients compared to controls (*P* = 0.001) [14]. Researchers have shown that M30 CK-18 and MMP-2 concentrations were higher in patients with CHB compared to healthy controls (*P* = 0.001 and *P* = 0.001, resp.) and were associated with significant liver fibrosis. What is worth emphasizing is that the assessment of MMP-2 levels did not show the difference between stages 1 and 2 as well stages 2 and 3 of liver fibrosis, highlighting the importance of labeling M30 CK-18 as a more sensitive marker than MMP-2 in predicting liver fibrosis [14]. In our study, we have shown not only the importance of the role of M30 CK-18 as a fibrosis marker but also the importance of M30 CK-18 as a good marker for inflammatory activity, which is of clinical importance for anti-HBV therapy indication, for example, in patients with high activity but with low fibrosis.

It is worth emphasizing that the determination of serum M30 CK-18 is more selective for the measurement of total CK-18 concentration. The measurement of the M30 CK-18 fragment reflects the hepatocytes apoptosis, which is a key process for the removal of infected cells in CHB. There are also other CK-18 fragments, such as the M65 epitope. In the study of Joka et al. [35], it was found that M65 was an additional indicator of a complementary M30, since it reflects the processes of necrosis. Reis et al. [36] in a study involving 76 patients with CHC after OLTx (orthotopic liver transplantation) measured M30S, M30H CK-18, and M65 CK-18 concentrations and found that these markers were able to discriminate between acute reinfection and acute transplant rejection (*P* = 0.048, *P* = 0.001, and *P* = 0.010). Only few studies have evaluated both fragments of M30 and M65-CK 18 in CHB. Zheng et al. found that the M30/M65 CK-18 ratio was statistically significantly higher in the CHB compared to that of the control group and highest in patients with acute chronic liver failure. The combination of these two markers had an AUC of ≥0.80 in the identification of liver failure in patients with CHB, which underlines the markedly marked M65 as a marker for hepatic necrosis [37]. Further work is needed to assess the usefulness of the measurement of CK-18 M56 patients with CHB hepatic failure.

The assessments of liver fibrosis and inflammatory activity are equally important for the clinical evaluation of liver function in chronic HBV infection. Since there are many ultrasound-based and serological noninvasive techniques that allow to evaluate liver fibrosis, there is an obvious need for noninvasive markers of inflammatory activity in the liver, especially in chronic HBV infection. According to EASL guidelines, the active inflammation in the liver in HBeAg(−) patients with HBV-DNA > 2000 U/L is an indication for the antiviral treatment. EASL 2017 Clinical Practice Guidelines allow the use of noninvasive biomarker markers, while do not specify which of them could be used [38]. In our study, we show that serum M30 CK-18 has better accuracy for the evaluation of liver inflammatory activity compared to ALT, while it is simple to use, does not require complex calculation algorithms, and is cheaper compared to commercial ones.

In conclusion, our study showed that serum concentration of M30 CK-18 reflects not only the advancement of fibrosis but also activity of disease. Serum M30 CK-18 > 253U/L has good sensitivity and specificity in discriminating mild versus moderate/severe fibrosis but also active liver inflammation even in patients with normal ALT activity. This study suggests M30 CK-18 as a potential noninvasive marker of CHB activity with clinical advantage compared with ALT activity but also facilitating the recognition the phase of disease.

## Figures and Tables

**Table 1:** Characteristics of studied population (median, IQR). Significant differences between consecutive phases by Kruskall-Wallis ANOVA were marked as follows: ^∗^*P* value < 0.05 and ^∗∗∗^*P* < 0.005.

	Total group (*n* = 150)	HBeAg(+) hepatitis (*n* = 10)	Low replicative carriers (*n* = 47)	High replicative carriers (*n* = 28)	HBeAg(−) hepatitis (*n* = 65)
Gender, M *n* (%)	86 (57)	7 (70)	19 (40)	15 (53)	45 (69)
Age, years	33 (23–42)	39 (24–53)	34 (25–45)	30 (23–39)	32 (23–40)
HBeAg(+), *n* (%)	10 (7)	10 (100)	—	—	—
HBV-DNA log10 IU/mL	4.1 (2.5–5.1)	5.1 (4.7-8.0)	2.3 (2.3–2.4)	3.9 (3.5–4.3)	5.0 (4.4-5.7)^∗∗∗^
HBsAg log10 IU/mL	4.2 (3.6–4.6)	4.3 (3.9–5.0)	4.1 (3.3-4.6)	4.1 (3.6–4.6)	4.3 (4.1–4.6)
ALT, IU/mL	30 (22–44)	70 (43–88)	23 (18–31)	27 (23–33)	36 (26–73)^∗∗∗^
GGT, IU/mL	18 (11–32)	28 (22–41)	13 (10–20)	18 (13–21)	25 (12–47)^∗∗∗^
Platelets, 10^9^/L	190 (157–220)	122 (110–154)	189 (155–219)	196 (171–224)	193 (166–219)
Prothrombin ratio, %	100 (90–107)	89 (82–98)	103 (96–110)	102 (92–110)	96 (89–107)^∗^

**Table 2:** Correlations between serum M30 CK-18 (U/L) and liver function tests as well as HBV-DNA and HBsAg levels (*R* value by Spearman's rank test; *β* value by multiple regression; ^∗^*P* value < 0.05).

Parameter	*R* value	*P* value	Multiple regression *β* value, *P* value
Age, years	0.13	0.09	—
HBV-DNA, log10 IU/mL	0.26	0.02^∗^	*B* = 0.23, *P* = 0.05^∗^
HBsAg, log10 IU/mL	−0.04	0.6	—
ALT, IU/mL	0.28	0.004^∗^	*B* = 0.32, *P* = 0.002^∗^
GGT, IU/mL	0.24	0.005^∗^	*B* = 0.25, *P* = 0.016^∗^
Platelets, 10^9^/L	−0.18	0.05^∗^	*B* = −0.06, *P* = 0.41

**Table 3:** Serum M30 CK-18 (median ± IQR) concentrations with regard to inflammatory activity and fibrosis stage in liver biopsy.

	Inflammatory activity		Fibrosis advancement	
	G0–G2 (*n* = 45)	G3–G4 (*n* = 21)	S0–S1 (*n* = 46)	S2–S4 (*n* = 20)
Age, years	31 (25–38)	39 (21–59)	31 (25–38)	39 (23–51)
HBV-DNA, log10 IU/mL	4.46 (3.6–5.28)	4.00 (3.23–6.19)	4.46 (3.61–5.27)	4.00 (3.18–7.39)
HBsAg, log10 IU/mL	4.25 (3.94–4.59)	4.14 (3.95–4.77)	4.27 (3.99–4.61)	4.11 (3.95–4.57)
CK-18, U/L	124 (74–225)	466 (197–1268)	120 (74–201)	521 (272–1268)
ALT, IU/mL	34 (26–52)	73 (30–109)	34 (27–51)	73 (36–109)
GGT, IU/mL	19 (12–32)	38 (28–114)	19 (12–32)	38 (28–114)
Platelets, 10^9^/L	198 (170–219)	127 (107–201)	201 (170–219)	126 (107–164)
